# Spatial segregation of piriform output neurons toward cognitive and emotional networks

**DOI:** 10.1093/pnasnexus/pgag026

**Published:** 2026-02-12

**Authors:** Chien-Fu F Chen, Donald A Wilson

**Affiliations:** Graduate Institute of Life Sciences, College of Biomedical Sciences, National Defense Medical University, No. 161, Sec. 6, Minquan E. Rd, Neihu Dist, Taipei City 114201, Taiwan; Department of Child and Adolescent Psychiatry, New York University Grossman School of Medicine, 1 Park Avenue 7th floor, New York, NY 10016, USA

**Keywords:** piriform cortex, odor perception, amygdala, prefrontal cortex, orbitofrontal cortex

## Abstract

The piriform cortex (PCx), commonly considered to be the primary olfactory sensory cortex, differs from other mammalian sensory cortices by not displaying a stimulus-specific spatial organization but rather displaying widely distributed odor-evoked activity. However, there is evidence of a PCx spatial organization based on output neuron targeting. Here, we performed double-labeled retrograde tracing to reveal neuronal populations of PCx output neurons that project to two regions affiliated with different behavioral significance, the basolateral amygdala (BLA) and lateral orbitofrontal (LO) cortex networks. We found that PCx neurons projecting to BLA and LO are distinct in spatial distribution with minimal overlap, supporting the hypothesis that while odor input is distributed randomly across the PCx, PCx output neurons are organized into target-specific neuronal populations that potentially serve as functional units for odor encoding and odor-guided behavior.

Significance Statement The piriform cortex (PCx), the largest component of the olfactory cortex, is hypothesized to play a key role in odor perception. An enigma of the PCx is its distributed odor-evoked neuronal patterns that lack a sensory-based spatial order. Recent work has begun to decipher these patterns using population coding models. However, a general characterization of such distributed populations and their function in driving odor-guided behavior remains lacking. Using retrograde tracing, we showed that PCx neurons projecting to the amygdala or prefrontal cortex are distinct in spatial distribution and nearly nonoverlapping. These target-defined neuronal populations have the potential to serve as odor encoding units, allowing parallel transmission of odor information to networks underlying distinct behavioral responses.

## Introduction

Olfaction allows an animal to detect external chemical cues and generate appropriate behaviors critical for survival, such as food seeking, predator avoidance, and intraspecific communication (1–3). These odor-guided behaviors can be driven by innate odor responses (4–6) or learned responses to odors (7–9). In the rodents, for example, several odorant receptors and neural substrates have been identified as critical for innate odor preference (10–13). These data support the labeled-line hypothesis that some odor signals (e.g. pheromones) may be processed by dedicated pathways toward effector circuits. However, other odor-evoked behaviors are the result of experience-induced changes both within and beyond the olfactory system (14–16).

In mammals, odors are processed by a three-level neural pathway composed of a transduction level (olfactory epithelium, OE), a primary process level (olfactory bulb, OB), and a sensory association level (olfactory cortex, OC). The OE contains millions of olfactory sensory neurons (OSNs) that perform odorant transduction via their solely expressed odorant receptor type (17–19). OSNs then deliver transduction results to spherical neuropils of the OB—glomeruli, which each receives convergent input from homogeneous OSNs (20–22) and is innervated by a set of interneurons and projection neurons that together shape the output of the OB (23). Beyond OB, odor signals are broadcasted by the glomerular projection neurons to multiple regions that are together termed OC (24, 25). What role each OC subregion specifically plays in olfaction is not entirely known; however, several common features have been identified. First, OC principal neurons are innervated by projection neurons of multiple glomeruli and excitable by multiglomerular stimulation (26–28), making them detectors for specific glomerular input combinations. Second, odors activate distributed and overlapping neuronal populations within OC regions (11, 29–31); these representations have been interpreted by various models of population coding (32–36). Third, OC regions are often directly connected to a larger network, including the ventral striatum (olfactory tubercle), hippocampus (lateral entorhinal cortex), and amygdaloid complex (37–39). These data together support the notion that OC regions are the odor-recognition level for larger associative networks (40). In this model, OC neuronal populations encode odors with distributed population codes, which are then decoded by their downstream target structures, driving specific behaviors. The behavioral outcomes of odor input to the OC are therefore definable by the combination of broadly distributed activities in the OC and the associated downstream region(s) (41–43). This cortical structure differs from other mammalian sensory systems by displaying a highly distributed organization of stimulus quality coding, in contrast to the stimulus-specific spatial coding (e.g. tonotopic or retinotopic) seen in other systems.

However, there is increasing evidence that OC, especially the piriform cortex (PCx), may display a form of spatial organization based not on sensory input, but rather on the efferent targeting (44). The PCx is known to project to multiple limbic and prefrontal centers (45, 46) and is ideal for retrograde tracing to reveal this target-dependent neuronal organization. In the present study, we further explored spatial patterns of PCx output connectivity toward behaviorally relevant centers. We found that PCx neurons projecting to the basolateral amygdala (BLA), which is involved in emotionally balanced behavioral response to odors, and the lateral orbitofrontal (LO) cortex, which is involved in the motivational significance of odors, are stereotypically organized, distinct in spatial distribution, and practically separated. These results provide anatomical grounds for PCx encoding units based on output and allow further examining models of OC population codes for odors and odor-guided behavior.

## Results

We performed retrograde tracing to reveal neuronal populations of the PCx that project to two regions affiliated with amygdaloid and orbitofrontal networks. We found that PCx neurons projecting to BLA and LO are distinct in spatial distribution and barely overlap, supporting the hypothesis that PCx neurons are organized into target-specific neuronal populations that potentially serve as functional units for odor encoding.

### Stereotyped and topographic distribution of BLA-projecting neurons of the PCx

**Fig. 1.**
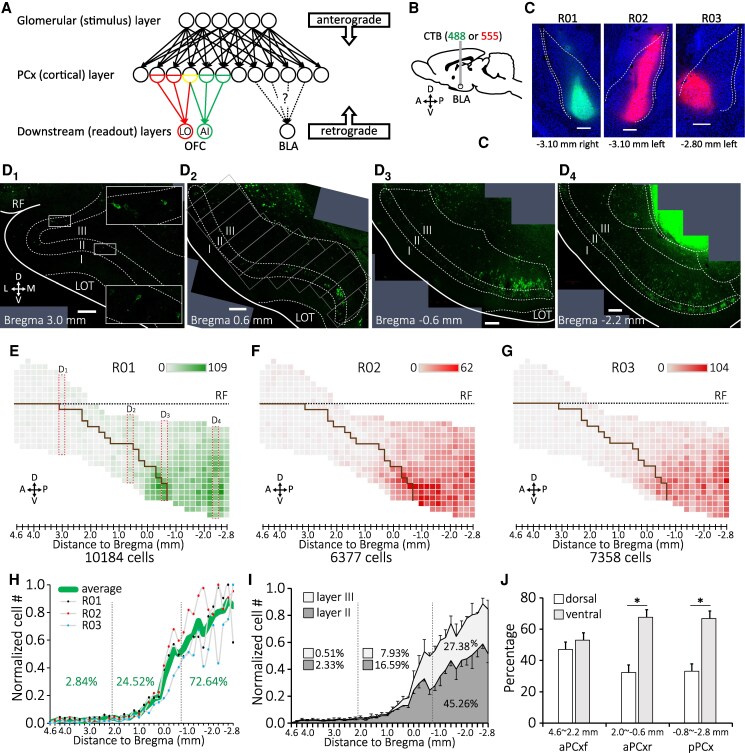
Topographic organization of BLA-projecting neurons of the PCx. A) Schematic description of PCx population units that are retrogradely defined by their targeting regions. B) CTB fluorescent conjugates used for the BLA retrograde tracing. C) The injection site of CTB in different animal subjects. Counterstain: NeuroTrace blue. D_1_–D_4_) PCx neurons labeled by CTB-488 on different coronal planes following the CTB injection into the BLA of animal R01. E–G) Distribution maps of layer II/III BLA-projecting neurons of the PCx for the three animals. Brown solid lines mark the dorsal edge of the LOT. Dashed horizontal lines mark the RF. H) Normalized cell count of layer II/III BLA-projecting neurons in the PCx along the A–P axis of the brains. Vertical dashed lines mark the boundaries between the PCx subdivisions. I) Average normalized cell counts of BLA-projecting neurons in layers II and III of the PCx along the A–P axis. J) Proportions of BLA-projecting neurons in dorsal and ventral portions of different PCx subdivisions.

The PCx has been the research focus of cortical odor processing. Its complex afferent and efferent connections can be comprehended via a simplified random feed-forward model (47, 48), where neurons receive random glomerular input while projecting to different targets, including the orbitofrontal cortex (OFC) and the BLA (Fig. 1A). It has been shown that PCx neurons projecting to subregions of the OFC are topographically and stereotypically distributed (44). This made us wonder how BLA-projecting neurons would distribute across the cortex. To answer this question, we injected retrograde tracer cholera toxin subunit B (CTB) with Alexa Fluor 488 or 555 into the left or right BLA of the rats (Figs. 1B, C and S1A–C). For example, CTB-488 was injected into the right BLA of rat R01 (Figs. 1C and S1A), resulting in green fluorescence in the BLA-projecting neurons (Fig. 1D_1–4_). We examined CTB-labeled cells in serial PCx sections that cover the entire anterior PCx (aPCx; sections with the lateral olfactory track, LOT) and a portion of posterior PCx (pPCx; sections without LOT) and found them predominately ipsilateral to the injection site and inclined to distribute in the ventro-posterior region of the PCx (Figs. 1D_1–4_ and S1D_1–4_, E_1–4_).

To further quantify neuronal distribution, we counted CTB-labeled cells in 200-μm-wide, layer II/III columns of the PCx aligned to the dorsal–ventral axis (Fig. 1D_2_). We then plotted the counting results into a standardized map, where each box represents a columnar cell count of a PCx section registered on the anterior-posterior (A–P) axis (Fig. 1E, F, G). Key anatomical landmarks, such as the rhinal fissure (RF; black dashed lines) and the LOT (brown solid lines) were marked to help identify PCx subregions. We found that, while different in total cell counts, the BLA-projecting neurons are similarly distributed across the animals. We observed a clear posterior preference of the BLA-projecting neurons in the line chart where the section-wise cell counts were normalized and plotted along the A–P axis (Fig. 1H). Here, we divided the aPCx into frontal aPCx (aPCxf, 4.6 to 2.2 mm anterior to bregma) and rear aPCx (aPCxr, 2.0 mm anterior to 0.6 mm posterior to bregma) depending on whether there is PCx buried within the RF (Fig. 1D_1_). On average, the BLA-projecting neurons are lowly distributed in the aPCxf (2.84% of total cells), moderately distributed in the aPCxr (24.52%), and heavily distributed in the pPCx (72.64%; Fig. 1H). The BLA-projecting neurons also exhibit distinct laminar distribution within each PCx subregion (Fig. 1I), with the ratios of layer III to layer II BLA-projecting neurons increasing from 0.22 (0.51%/2.33%) in the aPCxf to 0.48 (7.93%/16.59%) in the aPCxr to 0.60 (27.38%/45.26%) in the pPCx (Fig. 1I). In addition, as already noticed, we observed significant ventral preference of BLA-projecting neurons in the aPCxr (dorsal vs. ventral, 32.37% vs. 67.63%, *P* < 0.001) and the pPCx (33.13% vs. 66.87%, *P* < 0.001; Fig. 1J). These data together delineate the topographic distribution of BLA-projecting neurons of the PCx and support the notion that PCx output neurons are organized in a target-dependent manner (44).

### Distinct distribution between BLA- and LO-projecting neurons of the PCx

**Fig. 2.**
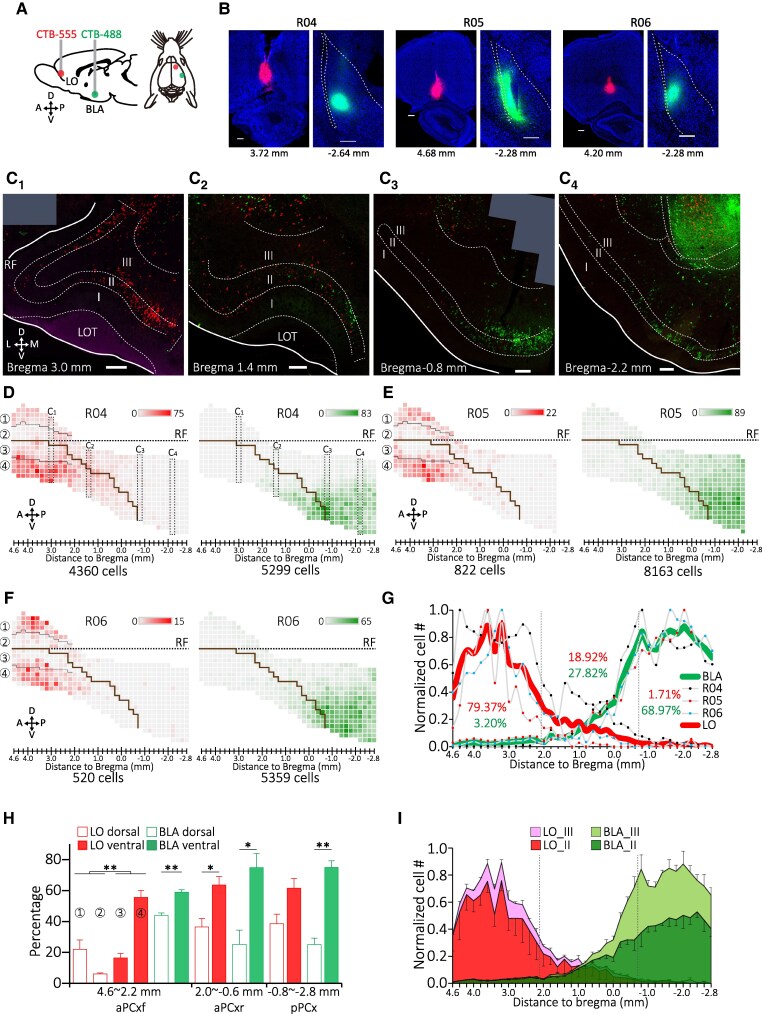
Distinct distribution of BLA- and LO-projection neurons of the PCx. A) Procedures of double retrograde tracing that involve injections of specific CTB conjugates into the BLA and LO of the right hemisphere. B) The injection site of CTB in different animal subjects. Counterstain: NeuroTrace blue. C_1_–C_4_) PCx neurons labeled by CTB-488 and CTB-555 on different coronal planes following the CTB injections into BLA and LO of animal R04. D–F) Distribution maps of layer II/III BLA-projecting neurons of the PCx for the three animals. Solid black lines mark the boundaries between the zones on the dorsal–ventral axis of aPCxf. G) Normalized cell count of layer II/III BLA- and LO-projecting neurons in the PCx along the A–P axis of the brains. H) Zonal distribution and dorsal–ventral preference of BLA- and LO-projecting neurons in different PCx subdivisions. I) Normalized laminar distribution of BLA- and LO-projecting neurons of the PCx along the A–P axis of the brain. *P < 0.05. **P < 0.01. Scale bars, 500 μm.

The distribution of the BLA-projecting neurons indicated that PCx output to the BLA relies on spatially biased populations of neurons. This result raised questions as to how neuronal populations with different targets distribute and overlap in the PCx. To address these questions, we conducted double retrograde tracing to reveal two target-specific neuronal populations in the PCx. We chose to compare the BLA-projecting neurons with the LO-projecting neurons, a population known to be concentrated in the aPCx (44). We injected CTB-488 into the BLA and CTB-555 into the LO of the same hemisphere of the rats (Fig. 2A and B), allowing BLA- and LO-projecting neurons to be differentially labeled in the PCx (Fig. 2C_1–4_).

We first examined the distribution of the BLA- and the LO-projecting neurons based on columnar cell counts plotted into the standardized map. These maps collectively reveal stereotyped distribution for either BLA- or LO-projecting neurons and distinct distribution patterns between these two populations (Fig. 2D–F). We found that both populations are biasedly distributed along A–P axis of the PCx, however in opposite directions (Fig. 2G). Furthermore, like BLA-projecting neurons, LO-projecting neurons exhibit ventral preference within the aPCx (aPCxf, dorsal vs. ventral, 28.03% vs. 71.97%, *P* = 0.0097; aPCxr, 36.45% vs. 63.55%, *P* = 0.026), with this preference trending toward significant in the pPCx (38.49% vs. 61.51%, *P* = 0.059; Fig. 2H). In fact, the LO-projecting neurons exhibit a zonal distribution such that neurons are mainly located in dorsal and ventral ends (zones 1 and 4) of the aPCxf (Fig. 2D–F and H). Another difference between the BLA- and the LO-projecting neuron is in their laminar distribution (Fig. 2I). On average, the BLA-projecting neurons are composed of 60.88% layer II and 39.12% layer III neurons, while the LO-projecting neurons are predominantly layer II neurons (83.12%). In addition, LO-projecting neurons remain lowly distributed in layer III across the PCx, but the percentage of layer III BLA-projecting neurons increases from 0.37% of the total population in aPCxf to 9.94% in aPCxr to 28.80% in pPCx. These results characterize the difference in distribution between two target-specific neuronal populations of the PCx, indicating a possibility that the PCx accommodates multiple population units that parallelly process odor signals for different extracortical targets.

**Fig. 3.**
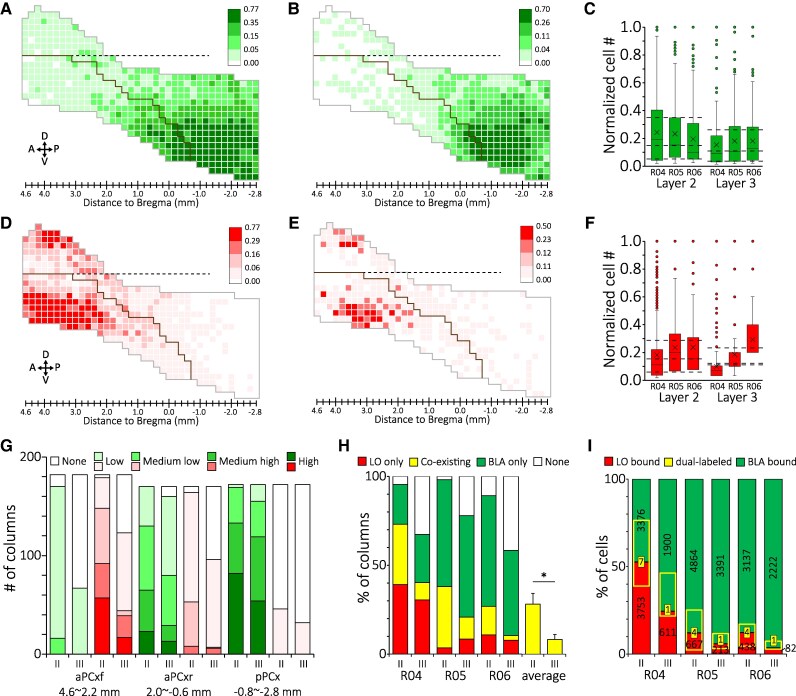
The extent of spatial and physical segregation between BLA- and LO-projecting neurons of the PCx. The average distribution maps of layer II (A) and layer III (B) BLA-projecting neurons from animals R04, R05, and R06. The color scales were defined by the average Q3, median, and Q1 of the population. C) Box plots of normalized cell count of BLA-projecting neurons in layer II and III columns of the PCx. Dashed lines marked the average Q3, median, and Q1 of the layer-specific population. The average distribution maps of layer II (D) and layer III (E) LO-projecting neurons from animals R04, R05, and R06. F. Box plots of normalized cell count of LO-projecting neurons in layer II and III columns of the PCx. Dashed lines mark the average Q3, median, and Q1 of the layer-specific population. G) Distribution of column types of the average distribution maps for BLA- and LO-projecting neurons in different PCx subdivisions. H) The extent of columnar overlapping of BLA- and LO-projecting neurons in different layers of the PCx. *P < 0.05. I) Physical overlapping of BLA- and LO-projecting neurons. Yellow rectangles mark the proportions of BLA- or LO-projecting neurons in the coexisting columns. The number in the yellow box indicates the number of dually labeled cells found in the layer-specific population of BLA- and LO-projecting neurons.

We noticed that BLA- and LO-projecting neurons appear to have different columnar distributions within the PCx (Fig. S2). To quantify this observation, we calculated normalized cell count for BLA- and LO-projecting neurons in each layer II and III column of the three animals. We then averaged these columnar cell counts to generate layer-specific distribution maps for the BLA-projecting (Fig. 3A and B) and the LO-projecting neurons (Fig. 3D and E). In these maps, the average numbers of Q3, median, and Q1 of the columnar cell distributions of the two neuronal populations were used to define four levels of columnar cell distribution: high, median high, median low, and low (Fig. 3C and F), and the columns were marked with different color scales in addition to white (no distribution for the population). Overall, these maps emphasize the inter-population differences mentioned earlier, including A–P preference, zonal distribution, and laminar preference. We then counted the number of distribution (white) columns in these maps to measure the distribution ranges of the two populations. For the BLA-projecting neurons, only 2.29% (12/524) of layer II and 23.86% (125/524) of layer III columns are white, and all these columns are in the aPCx (Fig. 3G). In contrast, the LO-projecting neurons are missing in 25.76% (135/524) of layer II and 52.10% (273/524) of layer III columns, with the majority (266/408) of these in the pPCx. These results confirm the histological observations and suggest differential participation of PCx subregions in ascending pathways toward OFC and amygdaloid networks.

### BLA- and LO-projecting neurons of the PCx are practically separated while coexisting in limited PCx columns

The striking difference in distribution between the BLA- and the LO-projecting neurons made us wonder how spatially segregated these two populations are. We addressed this question by examining how the BLA- and the LO-projecting neurons distribute within the layer II and III columns, which were categorized into LO only, BLA only, coexisting, and none (no distribution; Fig. 3H). We found that these three animals are diverse in the columnar composition, perhaps because of the difference in the injection sites and tracing results (Fig. 2B). However, certain features are shared among them. For example, more “None” columns are in layer III than layer II and yet more coexisting columns are in layer II than layer III of the PCx (Fig. 3H). On average, BLA- and LO-projecting neurons coexist in ∼28% of layer II and 8% of layer III columns of the PC (Fig. 3H); the neurons within coexisting columns account for 25% of layer II and 13% of layer III CTB-labeled cells of the PCx (Fig. 3I). With this level of spatial overlapping, surprisingly, physical overlapping between BLA- and LO-projecting neurons is barely seen, as indicated by the rare existence of dually labeled cells, which account for merely 0.09% of layer II and 0.04% of layer III neurons of the two populations combined (Fig. 3I). These results suggest that BLA- and LO-projecting neurons belong to different ascending pathways originating from the PCx.

## Discussion

The present results add to the growing evidence (44, 49) that the PCx displays a spatial organization strongly based on output neuron targeting rather than an odotopic sensory input. The results show that PCx output neurons are organized into target-specific neuronal populations, with LO-projecting neurons predominately located in layer II of aPCx and BLA-projecting neurons distributed across PCx and biasedly located in pPCx. Double-CTB-tracing experiments demonstrate nearly complete segregation of these two output neuronal populations at the cellular level, with fewer than 0.1% of individual neurons retrogradely labeled from both the LO and BLA. Combined with previous work demonstrating broadly distributed, nontopographic patterns of both OB input and PCx neuronal responses to odors (25, 28, 29, 31, 50) and recent population coding models (32–35), the present findings suggest that the PCx may be composed of multiple target-specific neuronal populations, which have access to similar (though not necessarily identical (51)) OB output with similar population codes for odor identity, but are different in downstream targets involved in different aspects of behavior.

A target-dependent organization has also been observed in Kenyon cells (KCs), third-order olfactory neurons of *Drosophila* (52, 53). KCs are classified based on their innervating lobes of the mushroom body (MB), where they form synapses with output and dopaminergic neurons in different MB compartments to allow the acquisition of odor-driven behaviors following associative learning (54–56). KCs have also been recognized as an analogy of piriform output neurons for their random glomerular innervation (57, 58) and distributed odor representation (59, 60). These interphylum similarities in sensory representation and higher olfactory connectivity raise an argument about the existence of a general neural solution that allows the animals to constantly adjust behaviors to new odor encounters and valences throughout life. Furthermore, the sequential development of the KC subpopulations may provide insight into how PCx neurons are organized into target-specific subpopulations during development (61).

The CTB fluorescent conjugates are widely used in neuronal tracing for their high accessibility, usability, and reliability (62–64). We noticed that CTB tracing is sensitive to A–P position of the injection sites, as we observed more LO-projection neurons in rear aPCxr and less BLA-projecting neurons in aPCxf of R04 (Fig. 2G), whose LO and BLA injection sites are relatively posterior to the other rats’ (Fig. 2B). CTB tracing is also sensitive to the laminar position of the tracer, as we observed fewer CTB-555 labeled neurons in aPCx of R06, whose CTB injection is relatively deeper into the LO (Fig. 2B). These factors contribute to individual differences in the columnar distribution of these two PCx output neuronal populations (Figs. 3H and S2).

It should be noted that the PCx, while being the primary source of olfactory input toward higher centers, also receives input from these centers, thus providing reciprocal feedback loops that appear to be differentially localized to PCx subregions (44, 65). For example, PCx output to the BLA includes both aPCxr and pPCx neurons, though with a ratio of about 1:3 (anterior:posterior; Fig. 1H), which matches well with BLA projection back to these subregions (66). In addition, while PCx-to-BLA projections mainly arise from the layer II neurons (Fig. 1I), BLA-to-PCx fibers largely innervate layers Ib and III of the pPCx (66, 67). Current and previous results suggest a similar reciprocal relationship and ascending–descending misalignment between the OFC and the aPCx (Fig. 2I) (68). These parallel feedback loops may contribute to different sensory/associative odor encoding between aPCx and pPCx during olfactory associative learning (16, 69) and allow PCx subregion output to be independently modulated by corresponding downstream feedback, likely in a state- or learning-dependent manner (70–72). Exploring the plasticity and sensory physiology of these output-target-defined populations of PCx neurons may provide insight into how the PCx contributes to the wide array of odor-dependent behavior.

## Materials and methods

### Animals, surgical procedures, and CTB retrograde tracing

We used adult Sprague Dawley rats (8–10 weeks old) purchased from a certified local vendor (BioLASCO Taiwan Co., Ltd) for all experiments. Upon arrival, the animals were housed on a 12-h light/dark cycle with food and water available ad libitum. All animal care and experimental protocols were conducted at National Defense Medical University (NDMU) and approved by the Institutional Animal Care and Use Committee. Fifty-six Sprague Dawley rats were used in this project, and 11 of them were considered successful for either single or double CTB injection. Tracing results of six rats (three single and three double CTB tracing) were further analyzed and presented in this study.

Before surgical procedures, anesthesia was conducted by intraperitoneal injection of Zotile/Xylazine cocktail (40 mg/10 mg/kg) into the animal. A deeply anesthetized animal was then placed into the stereotaxic device with a heating unit underneath its abdomen. To inject CTB, we conducted a craniotomy to create one or two burr holes (∼3 × 3 mm) on the dorsal surface of the skull, with dura mater carefully removed using a new 18 G needle. For the retrograde tracing, 200 nL of CTB solutions (0.5%, dissolved with phosphate buffer saline, Alexa Fuor 488 or 555 conjugates, C34775 or C34776; Thermo Fisher Scientific) were injected into LO (4.2 mm anterior, 2.3 mm lateral to bregma, 3.2 mm below surface) or BLA (2.4 mm posterior, 5.02 mm lateral to bregma, 7.7 mm below surface) using a 10-μL Hamilton syringe with a 33 G needle installed in the microinjector (TJ-2A, Longer Precision Pump Co., Ltd) at 10 nL/min.

### Histology, data acquisition, and analysis

Seven days after CTB injection, the animal was sacrificed and perfused with 4% paraformaldehyde. The fixed brain was collected and stored in a 30% sucrose solution for coronal cryosection. Brains were sectioned at 40-µm thickness into three or five copies ranging from 4.6 mm anterior to 2.8 mm posterior to the bregma. One copy of the sections was stained with NeuroTrace 435/455 Blue or 640/660 deep-red fluorescent Nissil stain (1:150 dilution) (N21479 or N21483, Thermo Fisher Scientific) and checked for CTB injection quality (e.g. location, size, etc.). The brain sections with good CTB injection were scanned into images using a ZEISS LSM 880 confocal microscope. Images were registered on the A–P axis using the rat brain atlas (73). To reveal the distribution of CTB-labeled cells in the PCx, cell counting was conducted in each 200-µm-wide layer II/III PCx column using ImageJ. Each neuronal distribution map of the PCx is composed of 38 images that were preregistered on the A–P axis, with each ∼200 µm apart. Results of the columnar cell counting were filled into Excel worksheets (Microsoft), which were then uploaded to Morpheus (https://software.broadinstitute.org/morpheus/) to generate the neuronal distribution maps. Statistics was performed using Student's t test.

## Supplementary Material

pgag026_Supplementary_Data

## Data Availability

All study data are included in the article and/or Supplementary material.

## References

[1] Doty RL . 1986. Odor-guided behavior in mammals. Experientia. 42:257–271. 3514263 10.1007/BF01942506

[2] Stowers L, Kuo TH. 2015. Mammalian pheromones: emerging properties and mechanisms of detection. Curr Opin Neurobiol. 34:103–109. 25747731 10.1016/j.conb.2015.02.005 PMC4561034

[3] Dulac C, O'Connell LA, Wu Z. 2014. Neural control of maternal and paternal behaviors. Science. 345:765–770. 25124430 10.1126/science.1253291 PMC4230532

[4] Holy TE . 2018. The accessory olfactory system: innately specialized or microcosm of mammalian circuitry? Annu Rev Neurosci. 41:501–525. 29727596 10.1146/annurev-neuro-080317-061916

[5] Silva BA, Gross CT, Gräff J. 2016. The neural circuits of innate fear: detection, integration, action, and memorization. Learn Mem. 23:544–555. 27634145 10.1101/lm.042812.116 PMC5026211

[6] Touhara K, Vosshall LB. 2009. Sensing odorants and pheromones with chemosensory receptors. Annu Rev Physiol. 71:307–332. 19575682 10.1146/annurev.physiol.010908.163209

[7] Li W, Wilson DA. 2024. Threat memory in the sensory cortex: insights from olfaction. Neuroscientist. 30:285–293. 36703569 10.1177/10738584221148994 PMC12707435

[8] Meissner-Bernard C, Dembitskaya Y, Venance L, Fleischmann A. 2019. Encoding of odor fear memories in the mouse olfactory cortex. Curr Biol. 29:367–380.e364. 30612908 10.1016/j.cub.2018.12.003

[9] Blass EM, Teicher MH. 1980. Suckling. Science. 210:15–22. 6997992 10.1126/science.6997992

[10] Demir E, et al 2020. The pheromone darcin drives a circuit for innate and reinforced behaviours. Nature. 578:137–141. 31996852 10.1038/s41586-020-1967-8

[11] Root CM, Denny CA, Hen R, Axel R. 2014. The participation of cortical amygdala in innate, odour-driven behaviour. Nature. 515:269–273. 25383519 10.1038/nature13897 PMC4231015

[12] Kobayakawa K, et al 2007. Innate versus learned odour processing in the mouse olfactory bulb. Nature. 450:503–508. 17989651 10.1038/nature06281

[13] Pérez-Gómez A, et al 2015. Innate predator odor aversion driven by parallel olfactory subsystems that converge in the ventromedial hypothalamus. Curr Biol. 25:1340–1346. 25936549 10.1016/j.cub.2015.03.026 PMC4439360

[14] Chen CF, Barnes DC, Wilson DA. 2011. Generalized vs. stimulus-specific learned fear differentially modifies stimulus encoding in primary sensory cortex of awake rats. J Neurophysiol. 106:3136–3144. 21918001 10.1152/jn.00721.2011 PMC3234083

[15] Wang PY, et al 2020. Transient and persistent representations of odor value in prefrontal cortex. Neuron. 108:209–224.e206. 32827456 10.1016/j.neuron.2020.07.033 PMC7886003

[16] Roesch MR, Stalnaker TA, Schoenbaum G. 2007. Associative encoding in anterior piriform cortex versus orbitofrontal cortex during odor discrimination and reversal learning. Cereb Cortex. 17:643–652. 16699083 10.1093/cercor/bhk009 PMC2396586

[17] Serizawa S, et al 2000. Mutually exclusive expression of odorant receptor transgenes. Nat Neurosci. 3:687–693. 10862701 10.1038/76641

[18] Firestein S . 1992. Electrical signals in olfactory transduction. Curr Opin Neurobiol. 2:444–448. 1525541 10.1016/0959-4388(92)90178-n

[19] Poivet E, et al 2016. Applying medicinal chemistry strategies to understand odorant discrimination. Nat Commun. 7:11157. 27040654 10.1038/ncomms11157 PMC4822015

[20] Mombaerts P, et al 1996. Visualizing an olfactory sensory map. Cell. 87:675–686. 8929536 10.1016/s0092-8674(00)81387-2

[21] Wang F, Nemes A, Mendelsohn M, Axel R. 1998. Odorant receptors govern the formation of a precise topographic map. Cell. 93:47–60. 9546391 10.1016/s0092-8674(00)81145-9

[22] Sullivan SL, Ressler KJ, Buck LB. 1995. Spatial patterning and information coding in the olfactory system. Curr Opin Genet Dev. 5:516–523. 7580145 10.1016/0959-437x(95)90057-n

[23] Kikuta S, Fletcher ML, Homma R, Yamasoba T, Nagayama S. 2013. Odorant response properties of individual neurons in an olfactory glomerular module. Neuron. 77:1122–1135. 23522047 10.1016/j.neuron.2013.01.022 PMC3607817

[24] Neville KR, Haberly L. Olfactory cortex. In: Shepherd GM, editor. The synaptic organization of the brain. Oxford University Press, New York, 2004. p. 415–454.

[25] Sosulski DL, Bloom ML, Cutforth T, Axel R, Datta SR. 2011. Distinct representations of olfactory information in different cortical centres. Nature. 472:213–216. 21451525 10.1038/nature09868 PMC3354569

[26] Davison IG, Ehlers MD. 2011. Neural circuit mechanisms for pattern detection and feature combination in olfactory cortex. Neuron. 70:82–94. 21482358 10.1016/j.neuron.2011.02.047 PMC3086570

[27] Apicella A, Yuan Q, Scanziani M, Isaacson JS. 2010. Pyramidal cells in piriform cortex receive convergent input from distinct olfactory bulb glomeruli. J Neurosci. 30:14255–14260. 20962246 10.1523/JNEUROSCI.2747-10.2010 PMC2972672

[28] Miyamichi K, et al 2011. Cortical representations of olfactory input by trans-synaptic tracing. Nature. 472:191–196. 21179085 10.1038/nature09714 PMC3073090

[29] Stettler DD, Axel R. 2009. Representations of odor in the piriform cortex. Neuron. 63:854–864. 19778513 10.1016/j.neuron.2009.09.005

[30] Kay RB, Meyer EA, Illig KR, Brunjes PC. 2011. Spatial distribution of neural activity in the anterior olfactory nucleus evoked by odor and electrical stimulation. J Comp Neurol. 519:277–289. 21165975 10.1002/cne.22519 PMC3342756

[31] Rennaker RL, Chen CF, Ruyle AM, Sloan AM, Wilson DA. 2007. Spatial and temporal distribution of odorant-evoked activity in the piriform cortex. J Neurosci. 27:1534–1542. 17301162 10.1523/JNEUROSCI.4072-06.2007 PMC2291208

[32] Bolding KA, Franks KM. 2017. Complementary codes for odor identity and intensity in olfactory cortex. Elife. 6:e22630. 28379135 10.7554/eLife.22630 PMC5438247

[33] Roland B, Deneux T, Franks KM, Bathellier B, Fleischmann A. 2017. Odor identity coding by distributed ensembles of neurons in the mouse olfactory cortex. Elife. 6:e26337. 28489003 10.7554/eLife.26337 PMC5438249

[34] Schaffer ES, et al 2018. Odor perception on the two sides of the brain: consistency despite randomness. Neuron. 98:736–742.e733. 29706585 10.1016/j.neuron.2018.04.004 PMC6026547

[35] Pashkovski SL, et al 2020. Structure and flexibility in cortical representations of odour space. Nature. 583:253–258. 32612230 10.1038/s41586-020-2451-1 PMC7450987

[36] Iurilli G, Datta SR. 2017. Population coding in an innately relevant olfactory area. Neuron. 93:1180–1197.e1187. 28238549 10.1016/j.neuron.2017.02.010 PMC5370575

[37] Cádiz-Moretti B, Abellán-Álvaro M, Pardo-Bellver C, Martínez-García F, Lanuza E. 2017. Afferent and efferent projections of the anterior cortical amygdaloid nucleus in the mouse. J Comp Neurol. 525:2929–2954. 28543083 10.1002/cne.24248

[38] Witter MP, Doan TP, Jacobsen B, Nilssen ES, Ohara S. 2017. Architecture of the entorhinal cortex A review of entorhinal anatomy in rodents with some comparative notes. Front Syst Neurosci. 11:46. 28701931 10.3389/fnsys.2017.00046 PMC5488372

[39] Wesson DW . 2020. The tubular striatum. J Neurosci. 40:7379–7386. 32968026 10.1523/JNEUROSCI.1109-20.2020 PMC7511186

[40] Wilson DA, Sullivan RM. 2011. Cortical processing of odor objects. Neuron. 72:506–519. 22099455 10.1016/j.neuron.2011.10.027 PMC3223720

[41] Kim J, Pignatelli M, Xu S, Itohara S, Tonegawa S. 2016. Antagonistic negative and positive neurons of the basolateral amygdala. Nat Neurosci. 19:1636–1646. 27749826 10.1038/nn.4414 PMC5493320

[42] Choi GB, et al 2011. Driving opposing behaviors with ensembles of piriform neurons. Cell. 146:1004–1015. 21925321 10.1016/j.cell.2011.07.041 PMC3230930

[43] Gore F, et al 2015. Neural representations of unconditioned stimuli in basolateral amygdala mediate innate and learned responses. Cell. 162:134–145. 26140594 10.1016/j.cell.2015.06.027 PMC4526462

[44] Chen CF, et al 2014. Nonsensory target-dependent organization of piriform cortex. Proc Natl Acad Sci U S A. 111:16931–16936. 25385630 10.1073/pnas.1411266111 PMC4250170

[45] Johnson DM, Illig KR, Behan M, Haberly LB. 2000. New features of connectivity in piriform cortex visualized by intracellular injection of pyramidal cells suggest that “primary” olfactory cortex functions like “association” cortex in other sensory systems. J Neurosci. 20:6974–6982. 10995842 10.1523/JNEUROSCI.20-18-06974.2000 PMC6772836

[46] Schwabe K, Ebert U, Löscher W. 2004. The central piriform cortex: anatomical connections and anticonvulsant effect of GABA elevation in the kindling model. Neuroscience. 126:727–741. 15183521 10.1016/j.neuroscience.2004.04.022

[47] Babadi B, Sompolinsky H. 2014. Sparseness and expansion in sensory representations. Neuron. 83:1213–1226. 25155954 10.1016/j.neuron.2014.07.035

[48] Haberly LB . 2001. Parallel-distributed processing in olfactory cortex: new insights from morphological and physiological analysis of neuronal circuitry. Chem Senses. 26:551–576. 11418502 10.1093/chemse/26.5.551

[49] White KA, et al 2019. Glutamatergic neurons in the piriform cortex influence the activity of D1- and D2-type receptor-expressing olfactory tubercle neurons. J Neurosci. 39:9546–9559. 31628176 10.1523/JNEUROSCI.1444-19.2019 PMC6880455

[50] Illig KR, Haberly LB. 2003. Odor-evoked activity is spatially distributed in piriform cortex. J Comp Neurol. 457:361–373. 12561076 10.1002/cne.10557

[51] Nagayama S, et al 2010. Differential axonal projection of mitral and tufted cells in the mouse main olfactory system. Front Neural Circuits. 4:120. 20941380 10.3389/fncir.2010.00120 PMC2952457

[52] Crittenden JR, Skoulakis EM, Han KA, Kalderon D, Davis RL. 1998. Tripartite mushroom body architecture revealed by antigenic markers. Learn Mem. 5:38–51. 10454371 PMC311260

[53] Tanaka NK, Awasaki T, Shimada T, Ito K. 2004. Integration of chemosensory pathways in the *Drosophila* second-order olfactory centers. Curr Biol. 14:449–457. 15043809 10.1016/j.cub.2004.03.006

[54] Aso Y, et al 2014. The neuronal architecture of the mushroom body provides a logic for associative learning. Elife. 3:e04577. 25535793 10.7554/eLife.04577 PMC4273437

[55] Cognigni P, Felsenberg J, Waddell S. 2018. Do the right thing: neural network mechanisms of memory formation, expression and update in Drosophila. Curr Opin Neurobiol. 49:51–58. 29258011 10.1016/j.conb.2017.12.002 PMC5981003

[56] Owald D, et al 2015. Activity of defined mushroom body output neurons underlies learned olfactory behavior in *Drosophila*. Neuron. 86:417–427. 25864636 10.1016/j.neuron.2015.03.025 PMC4416108

[57] Caron SJ, Ruta V, Abbott LF, Axel R. 2013. Random convergence of olfactory inputs in the *Drosophila* mushroom body. Nature. 497:113–117. 23615618 10.1038/nature12063 PMC4148081

[58] Hayashi TT, et al 2022. Mushroom body input connections form independently of sensory activity in *Drosophila melanogaster*. Curr Biol. 32:4000–4012.e4005. 35977547 10.1016/j.cub.2022.07.055 PMC9533768

[59] Wang Y, et al 2001. Genetic manipulation of the odor-evoked distributed neural activity in the *Drosophila* mushroom body. Neuron. 29:267–276. 11182097 10.1016/s0896-6273(01)00196-9

[60] Campbell RA, et al 2013. Imaging a population code for odor identity in the *Drosophila* mushroom body. J Neurosci. 33:10568–10581. 23785169 10.1523/JNEUROSCI.0682-12.2013 PMC3685844

[61] Lee T, Lee A, Luo L. 1999. Development of the *Drosophila* mushroom bodies: sequential generation of three distinct types of neurons from a neuroblast. Development. 126:4065–4076. 10457015 10.1242/dev.126.18.4065

[62] Conte WL, Kamishina H, Reep RL. 2009. Multiple neuroanatomical tract-tracing using fluorescent Alexa Fluor conjugates of cholera toxin subunit B in rats. Nat Protoc. 4:1157–1166. 19617887 10.1038/nprot.2009.93

[63] Diodato A, et al 2016. Molecular signatures of neural connectivity in the olfactory cortex. Nat Commun. 7:12238. 27426965 10.1038/ncomms12238 PMC4960301

[64] Altshuler RD, et al 2025. Profiling gene alterations in striatonigral neurons associated with incubation of methamphetamine craving by cholera toxin subunit B-based fluorescence-activated cell sorting. Front Cell Neurosci. 19:1542508. 40012565 10.3389/fncel.2025.1542508 PMC11860961

[65] Wang L, et al 2020. Cell-type-specific whole-brain direct inputs to the anterior and posterior piriform cortex. Front Neural Circuits. 14:4. 32116571 10.3389/fncir.2020.00004 PMC7019026

[66] Majak K, Rönkkö S, Kemppainen S, Pitkänen A. 2004. Projections from the amygdaloid complex to the piriform cortex: a PHA-L study in the rat. J Comp Neurol. 476:414–428. 15282713 10.1002/cne.20233

[67] Luna VM, Morozov A. 2012. Input-specific excitation of olfactory cortex microcircuits. Front Neural Circuits. 6:69. 23049500 10.3389/fncir.2012.00069 PMC3446699

[68] Illig KR . 2005. Projections from orbitofrontal cortex to anterior piriform cortex in the rat suggest a role in olfactory information processing. J Comp Neurol. 488:224–231. 15924345 10.1002/cne.20595 PMC1360190

[69] Calu DJ, Roesch MR, Stalnaker TA, Schoenbaum G. 2007. Associative encoding in posterior piriform cortex during odor discrimination and reversal learning. Cereb Cortex. 17:1342–1349. 16882682 10.1093/cercor/bhl045 PMC2473864

[70] Cohen Y, Reuveni I, Barkai E, Maroun M. 2008. Olfactory learning-induced long-lasting enhancement of descending and ascending synaptic transmission to the piriform cortex. J Neurosci. 28:6664–6669. 18579740 10.1523/JNEUROSCI.0178-08.2008 PMC6670420

[71] Lo H, et al 2025. Feeding-induced olfactory cortex suppression reduces satiation. Neuron. 113:2856–2871.e8. 40818450 10.1016/j.neuron.2025.07.020

[72] Sadrian B, Wilson DA. 2015. Optogenetic stimulation of lateral amygdala input to posterior piriform cortex modulates single-unit and ensemble odor processing. Front Neural Circuits. 9:81. 26733819 10.3389/fncir.2015.00081 PMC4685079

[73] Paxinos G, Watson C. The rat brain in stereotaxic coordinates. 6th ed. Academic Press/Elsevier, Amsterdam, Boston, 2007.

