# Supplementary material for: Spatial segregation of piriform output neurons toward cognitive and emotional networks
Source: PNAS Nexus. 2026 Feb 12;5(2):pgag026. doi: 10.1093/pnasnexus/pgag026 (PMC12917545; doi:10.1093/pnasnexus/pgag026)
Supplement: pgag026_Supplementary_Data [file pgag026_supplementary_data.pdf]

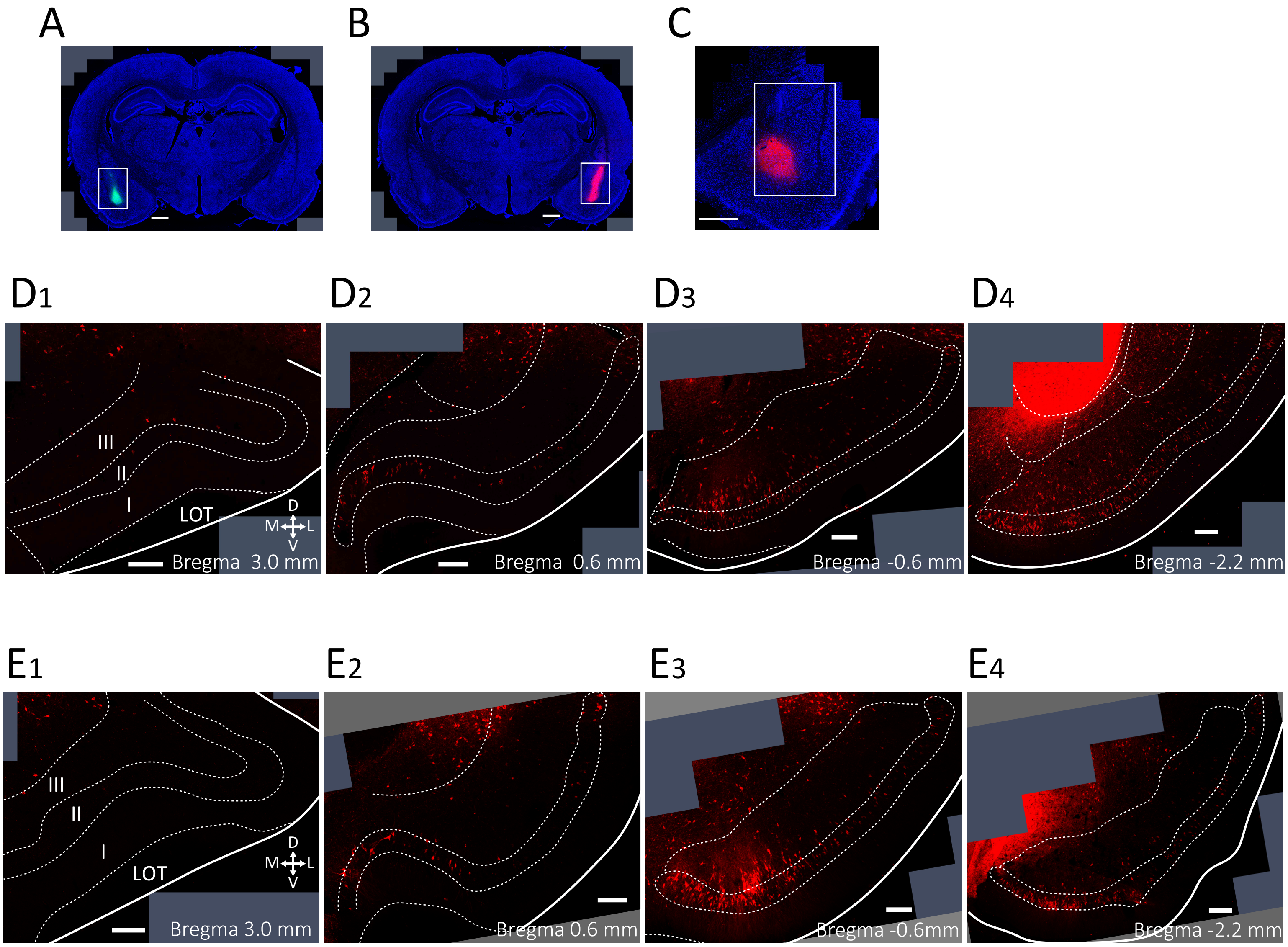

Figure S1. BLA-projecting neurons of the PCx revealed by CTB retrograde tracing. **A.** CTB-488 was injected into the right BLA (white box) of animal R01. CTB-555 was injected into left BLA (white box) of animal R02 (**B**) and animal R03 (**C**). **D1 to D4**. PCx neurons labled by CTB-555 on different coronal plans following the CTB injection into BLA of animal R02. **E1 to E4.** PCx neurons labled by CTB-555 on different coronal plans following the CTB injection into BLA of animal R03. Scale bars in **A**, **B** & **C**, 1000 µm; in **D** & **E**, 200 µm.

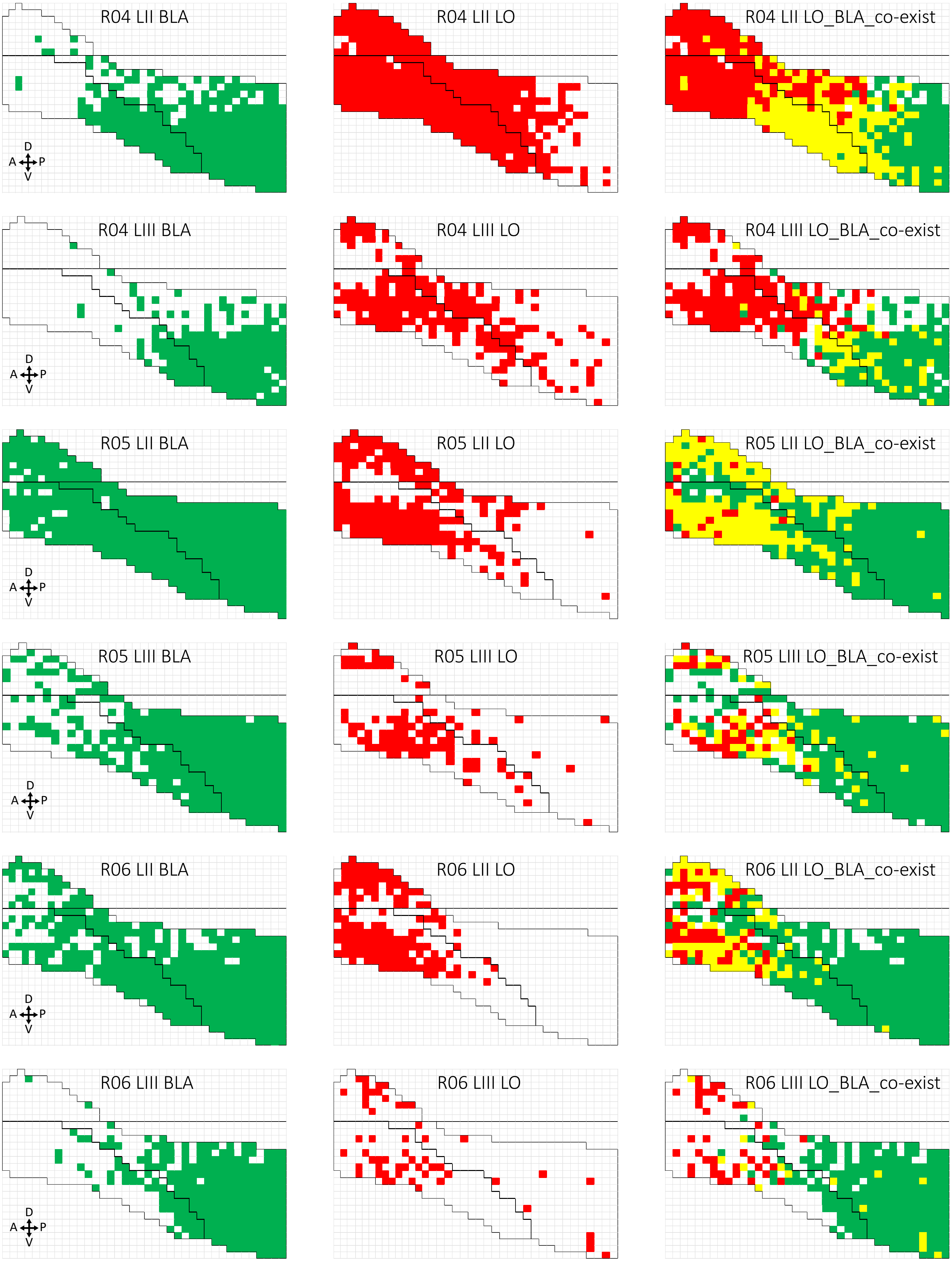

Figure S2. Columnar distribution of BLA- and LO-projecting neurons of the PCx. Left column. Layer-specific distribution of PCx columns of three animals containing BLA-projecting neurons (green boxes). Middle column. Layer-specific distribution of the PCx columns containing LO-projecting neurons (red boxes). Right column. Layer-specific distribution of the PCx columns containing only BLA-projecting neurons (green boxes), only LO-projecting neurons (red boxes), and both populations (yellow boxes). White boxes indicate the PCx columns where niehter BLA- nor LO-projecting neurons were identified.
